# Supplementary material for: 4D nucleome equation predicts gene expression controlled by long-range enhancer-promoter interaction
Source: PLoS Comput Biol. 2023 Dec 18;19(12):e1011722. doi: 10.1371/journal.pcbi.1011722 (PMC10760824; doi:10.1371/journal.pcbi.1011722)
Supplement: S1 Text — In the first section, we supplement the details of the model and give the simulation algorithm as well as the statistics analyses of the simulation data. In the second section, we present the procedure for fitting experimental data using our model and comparing the experimental data with theoretical results. (PDF) [file pcbi.1011722.s001.pdf]

4D nucleome equation predicts gene expression controlled by long-range enhancer-promoter interaction

Zihao Wang[1,2], Songhao Luo[1,2], Zhenquan Zhang[1,2], Tianshou Zhou[1,2,*], and Jiajun Zhang[1,2,*]

[1] Guangdong Province Key Laboratory of Computational, Sun Yat-sen University, Guangzhou 510275, P. R. China

[2] School of Mathematics, Sun Yat-Sen University, Guangzhou 510275, P. R. China

* Corresponding author

Email: mcszhtsh@mail.sysu.edu.cn (TZ), zhjiajun@mail.sysu.edu.cn (JZ)

Contents

I. Model Description and Simulation **3**

A. Model details 3

B. Simulation details 4

C. Statistics analyses of the simulated data 5

D. Theoretical analysis of E-P spatial distance 5

II. Fitting Experimental Data Using Model **6**

A. Preliminary attempt 6

B. Parameter estimation of gene expression dynamics 6

C. Comparing experimental data with theoretical results 8

D. Discussing with Zuin's model 8

E. Importance of slow chromatin dynamics 9

Reference **11**

This supplementary information contains three sections. In the first section, we supplement the details of the model, give the simulation algorithm and the statistics analyses of the simulation data, and present the derivation of E-P spatial distance distribution. In the second section, we present the procedure for fitting experimental data using our model and comparing the experimental data with theoretical results.

I. Model Description and Simulation

A. Model details

The upstream evolution based on Langevin equation can be rewritten as

$$\mathrm{d}\boldsymbol{r} = -\frac{1}{\gamma}\left[k_{\mathrm{NN}}\boldsymbol{M}_{\mathrm{NN}} + k_{\mathrm{EP}}\boldsymbol{M}_{\mathrm{EP}}\right]\boldsymbol{r}\mathrm{d}t + \sqrt{2D}\mathrm{d}\boldsymbol{B}(t), \quad [1]$$

where $\boldsymbol{M}_{\mathrm{NN}}$ and $\boldsymbol{M}_{\mathrm{EP}}$ are $N \times N$ matrixes showing the connectivity between adjacent beads and the E-P interaction respectively.

$$\boldsymbol{M}_{\mathrm{NN}}^{i,j} = \begin{cases} -\sum_{l \neq i} \boldsymbol{M}_{\mathrm{NN}}^{i,l}, & i = j, \\ -1, & |i-j| = 1, \\ 0, & otherwise. \end{cases} \quad \boldsymbol{M}_{\mathrm{EP}}^{i,j} = \begin{cases} -\sum_{l \neq i} \boldsymbol{M}_{\mathrm{EP}}^{i,l}, & i = j, \\ -1, & (i,j) = (\mathrm{E},\mathrm{P}) \text{ or } (i,j) = (\mathrm{P},\mathrm{E}), \\ 0, & otherwise. \end{cases} \quad [2]$$

We neglect attraction or repulsion between other monomers in the chain.

The downstream mRNA number can reach to infinity theoretically, so the number of gene state K is infinite. Thus, the state transition matrix of gene expression reactions is an infinite dimension matrix.

The state-switching rate vector $\boldsymbol{\theta}$ depends nonlinearly on the E-P spatial distance d_{S}. Thus, the two-state model becomes a variable two-state model characterized by $\boldsymbol{\theta} = \boldsymbol{H}(d_{\mathrm{s}})$. Taking the variable α as an example, the variable α depending on the E-P spatial distance is

$$\alpha(d_{\mathrm{S}}) = \begin{cases} \alpha_{\max}, & d_{\mathrm{S}} \le b, \\ \alpha_{\min} + (\alpha_{\max} - \alpha_{\min}) \Big/ \left[1 + \left(\dfrac{d_{\mathrm{S}} - b}{b_{1/2} - b}\right)^h\right], & d_{\mathrm{S}} > b, \end{cases} \quad [3]$$

where $\alpha_{\min}$ and $\alpha_{\max}$ are the minimum (basic) and maximum reaction rates, $b_{1/2}$ is the spatial distance when $\alpha(d_{\mathrm{S}})$ is equal to $(\alpha_{\max} - \alpha_{\min})/2$, h is a Hill coefficient that controls how steep the rate curve is, and b is a distance threshold. When the E-P distance is less than b, the b is also used to describe the encounter of E-P. Therefore, it is reasonable to assume that the state transition rates are maximum when the distance is less than b. Besides, encounter, which is merely an assumption in physical statistics, does not mean direct contact. As the distance increases, state transition rates can reduce to the minimum. We may also choose a linear dependence of the activation rate on E-P contacts at an appropriate distance, that is

$$\alpha(d_{\mathrm{S}}) = \begin{cases} \alpha_{\min} + \dfrac{\alpha_{\max} - \alpha_{\min}}{b'} d_{\mathrm{S}}, & d_{\mathrm{S}} \le b', \\ \alpha_{\min}, & d_{\mathrm{S}} > b', \end{cases} \quad [4]$$

where b' is used to describe a large spatial distance between enhancer and promoter that the gene is basically in the inactive state.

B. Simulation details

The model we used couples a two-state gene expression model to the three-dimensional kinetics of chromatin motion. In fact, if we only consider the chromatin dynamics, the time evolution of the chromatin (especially E-P interaction) is based on the Langevin equation (Eq. [1]). If we only consider the gene expression, its dynamics is based on a master equation approach, and use the Gillespie algorithm to simulate it. However, due to the coupling of the E-P interaction and gene expression, we propose an algorithm to simulate the time evolution of the entire system.

The spatial positions of nucleosomes in 3D space are denoted by $\boldsymbol{r}$. There are four reactions involved in the gene-state switching process. The l th reaction propensity is denoted by $a_l(\boldsymbol{r},\boldsymbol{s};t)$, $l=1,2,3,4$, and the total reaction propensity is calculated according to $a_{\text{tot}}(\boldsymbol{r},\boldsymbol{s};t)=\sum_{l=1}^{4}a_l(\boldsymbol{r},\boldsymbol{s};t)$. Note that $\boldsymbol{s}$ is the vector of all gene states. Element v_{ij} of stoichiometric matrix $(v_{ij})_{K\times L}$ denotes the net change in gene state j due to each reaction i, and $\boldsymbol{v}_\mu$ is the μ th column of (v_{ij}). Let $H(\boldsymbol{r},\boldsymbol{s};t)$ is the survival probability that chromatin position is $\boldsymbol{r}$ and gene state is $\boldsymbol{s}$ state at time t. The main steps are listed below:

(1) Set the initial state as $\boldsymbol{r}_0=\boldsymbol{r}(t_0),\ \boldsymbol{s}_0=\boldsymbol{s}(t_0)$.

(2) Generate two random variables u_1 and u_2 distributed uniformly in interval $(0,1)$.

(3) Integrate the system of stochastic differential equations

$$\begin{cases}\mathrm{d}\boldsymbol{r}=\boldsymbol{V}(\boldsymbol{r};t)\mathrm{d}t+\sqrt{2\boldsymbol{D}}\mathrm{d}\boldsymbol{B}(t),\\ \mathrm{d}H(\boldsymbol{r},\boldsymbol{s};t)=-a_{\text{tot}}(\boldsymbol{r},\boldsymbol{s};t)H(\boldsymbol{r},\boldsymbol{s};t)\mathrm{d}t,\\ \boldsymbol{r}(t_i)=\boldsymbol{r}_i,\ H(t_i)=1,\end{cases} \tag{5}$$

from time points t_i to $t_i+\tau_i$, and with the stopping condition $H(\boldsymbol{r}_i,\boldsymbol{s}_i;t_i+\tau_i)=u_1$.

(4) Update time and position: $t_{i+1}=t_i+\tau_i$, $\boldsymbol{r}_{i+1}=\boldsymbol{r}(t_{i+1})$.

(5) Choose μ such that

$$\sum_{l=1}^{\mu-1}a_l(\boldsymbol{r}_{i+1},\boldsymbol{s}_i;t_{i+1})<u_2a_{\text{tot}}(\boldsymbol{r}_{i+1},\boldsymbol{s}_i;t_{i+1})\le\sum_{l=1}^{\mu}a_l(\boldsymbol{r}_{i+1},\boldsymbol{s}_i;t_{i+1}). \tag{6}$$

(6) Update promoter state: $\boldsymbol{s}_{i+1}=\boldsymbol{s}_i+\boldsymbol{v}_\mu$.

(7) Reiterate the system from step (2) with a new state until a given largest time $t_{\max}$ is reached.

By the above algorithm steps, we can generate sample trajectories of the system. In the simulation, we examine a system consisting of $N=100$ monomers in simulations. To simplify, we consider a spatial region isolated from neighboring DNA by boundary insulator elements (1, 2).

For a given set of parameters, we simulate 10^3 gene copies, proceeding through 10^7 seconds in time. Each simulation starts in the OFF state. Before taking samples from every simulation, we run 10^4 seconds to ensure equilibration in chromatin conformation and gene expression. After that, snapshots of the system are taken every 100 seconds.

C. Statistics analyses of the simulated data

We can obtain the time series of chromatin structure (especially E-P spatial distance) and gene-state switching as well as mRNA level by simulation. Therefore, we statistically perform the probability density function of E-P spatial distance and probability mass function of mRNA level. Furthermore, we calculate the statistical quantities such as mRNA mean level and coefficient of variation (CV).

D. Theoretical analysis of E-P spatial distance

We derive the probability density function (PDF) of the E-P spatial distance distribution. Term $k_{\mathrm{NN}}\boldsymbol{M}_{\mathrm{NN}}+k_{\mathrm{EP}}\boldsymbol{M}_{\mathrm{EP}}$ in Eq. [1] is a singular matrix. To eliminate the degrees of freedom, the position of the first monomer in the chain can be set as $\boldsymbol{r}_1\equiv\boldsymbol{0}$ based on the methods in (3). Thus, the Fokker-Planck equation corresponding to Eq. [1] can be rewritten as

$$\frac{\partial p(\tilde{\boldsymbol{r}};t)}{\partial t}=\nabla_{\tilde{\boldsymbol{r}}}\cdot\left(\frac{1}{\gamma}\left[k_{\mathrm{NN}}\tilde{\boldsymbol{M}}_{\mathrm{NN}}+k_{\mathrm{EP}}\tilde{\boldsymbol{M}}_{\mathrm{EP}}\right]\tilde{\boldsymbol{r}}p(\tilde{\boldsymbol{r}};t)\right)+\nabla_{\tilde{\boldsymbol{r}}}^2\left(Dp(\tilde{\boldsymbol{r}};t)\right), \tag{7}$$

where $\tilde{\boldsymbol{r}}=(r_{ij})_{(N-1)\times 3}=[\boldsymbol{r}_2,\ \cdots,\ \boldsymbol{r}_N]^{\mathrm{T}}$, and $\tilde{\boldsymbol{M}}_{\mathrm{NN}}$ and $\tilde{\boldsymbol{M}}_{\mathrm{EP}}$ are $(N-1)\times(N-1)$ matrixes satisfying

$$\boldsymbol{M}_{\mathrm{NN}}=\left[\begin{array}{c|c}1 & \\ \hline & \tilde{\boldsymbol{M}}_{\mathrm{NN}}\end{array}\right],\ \boldsymbol{M}_{\mathrm{EP}}=\left[\begin{array}{c|c}0 & \\ \hline & \tilde{\boldsymbol{M}}_{\mathrm{EP}}^{i,j}\end{array}\right]. \tag{8}$$

Owing to the fact that every monomer moves independently in each dimension, the PDF for chromatin conformation $\tilde{\boldsymbol{r}}$ can be expressed as

$$p(\boldsymbol{r}_2,\cdots,\boldsymbol{r}_N)=\prod_{i=1}^{3}p_i(r_{2i},\cdots,r_{Ni}). \tag{9}$$

In fact, the monomer motion in Eq. [7] is an Ornstein-Uhlenbeck process and the general solution to this equation is a Gaussian distribution. If we consider one-dimensional PDF p_i, the p_i can be analytically expressed as (4)

$$p_i(r_{2i},\cdots,r_{Ni})=C\exp\left(-\frac{1}{2}(r_{2i},\cdots,r_{Ni})^{\mathrm{T}}\boldsymbol{\Sigma}^{-1}(r_{2i},\cdots,r_{Ni})\right), \tag{10}$$

where C is a normalization constant and

$$\boldsymbol{\Sigma}_{(N-1)\times(N-1)}^{-1}=\frac{1}{D\gamma}\left(k_{\mathrm{NN}}\tilde{\boldsymbol{M}}_{\mathrm{NN}}+k_{\mathrm{EP}}\tilde{\boldsymbol{M}}_{\mathrm{EP}}\right). \tag{11}$$

The p_i is a multivariate normal distribution with the zero mean and the covariance matrix being $\boldsymbol{\Sigma}$. The marginal distributions for the enhancer and promoter are calculated according to

$$\begin{aligned}p_i(r_{\mathrm{E}i})&=\int\cdots\int p_i(r_{2i},\cdots,r_{Ni})\prod_{l=2,\ l\neq \mathrm{E}}^{N}\mathrm{d}r_{li},\\ p_i(r_{\mathrm{P}i})&=\int\cdots\int p_i(r_{2i},\cdots,r_{Ni})\prod_{l=2,\ l\neq \mathrm{P}}^{N}\mathrm{d}r_{li}.\end{aligned} \tag{12}$$

Based on the properties of Gaussian distribution, the marginal PDFs in Eq. [12] are also Gaussian distributions.

By calculating, we find that the analytical expression of the PDF $p_{DS}\left(d_S\right)$ of the E-P spatial distance takes the form

$$p_{DS}\left(d_S\right)=\sqrt{\frac{2}{\pi}}\Theta^{-3}d_S^2\exp\left(-\frac{d_S^2}{2\Theta^2}\right), \quad [13]$$

where

$$\Theta=\sqrt{D\gamma\left(\frac{k_{NN}}{d_G}+k_{EP}\right)^{-1}}. \quad [14]$$

In Eq. [14], d_G is the E-P genomic distance, i.e., $d_G = P - E$. Note that Eq. [13] is a Maxwell-Boltzmann distribution, which can be regarded as the positive square root of the sum of squares of three independent random variables with each following the same normal distribution. Equivalently, each normal distribution represents the E-P distance in a certain dimension. Thus, Eq. [13] is the distribution of the E-P Euclidean distance in 3D. More precisely, Eq. [13] is equivalent to the chi distribution with three degrees of freedom and a scale parameter Θ.

II. Fitting Experimental Data Using Model

A. Preliminary attempt

We ignore the E-P regulatory and only use the two-state model to infer gene expression dynamics in different cell lines C_k ($k=1,...,6$). We define distinctive variable ON-OFF model. For example, the variable α two-state model is that only the α (ON) rates are distinctive and other parameters (β,μ,δ) are the same in different cell lines. That is the parameter $\mathbf{\Gamma}=\left[\alpha_1,\alpha_2,\alpha_3,\alpha_4,\alpha_5,\alpha_6,\beta,\mu\right]$. The variable α and μ two-state model is α and μ changes at the same time in different cell lines. Calculating the cross entropy (H) and minimizing the cross entropy (minH), we find the H reaches to minimum when the α and μ changes simultaneously in different cell lines (see Table A). Meanwhile, changing α and μ will affect the burst size and burst frequency theoretically, which is consistent with many experimental articles (5, 6).

Table A. Cross entropy minimization for different models that ignore long-range E-P regulatory.

**Model**	Variable α	Variable β	Variable μ
minH	11.6491	11.7762	11.9142
**Model**	Variable $\alpha+\beta$	Variable $\alpha+\mu$	Variable $\beta+\mu$
minH	13.4773	**11.5038**	11.7091

B. Parameter estimation of gene expression dynamics

Now, we consider the gene expression process controlled by long-range E-P interactions. Based on above results and the model assumption that the relationship between the α and E-P

distance may be opposite to that between β and E-P distance, we fixed the β rate. Therefore, we define three variable two-state models for transcription process: the variable α two-state model for which the parameter $\mathbf{\Gamma}=[k_{\mathrm{NN}},k_{\mathrm{EP}},\gamma,\alpha_{\min},\alpha_{\max},\beta,\mu]$, the variable μ two-state model for which $\mathbf{\Gamma}=[k_{\mathrm{NN}},k_{\mathrm{EP}},\gamma,\alpha,\beta,\mu_{\min},\mu_{\max}]$, the variable α and μ two-state model for which $\mathbf{\Gamma}=[k_{\mathrm{NN}},k_{\mathrm{EP}},\gamma,\alpha_{\min},\alpha_{\max},\beta,\mu_{\min},\mu_{\max}]$. And α and μ rate nonlinearly depends on E-P interaction through a Hill function Eq. [3].

We use the *fmincon* function (a nonlinear programming solver) in the LBFGS method of MATLAB to find the minimum value of the optimization problem given a set of initial values and parameter intervals. $k_{\mathrm{NN}}=(10^{-3},10)$, $k_{\mathrm{EP}}=(10^{-3},10)$, $\lambda=(1,10^{2})$, $\alpha_{\max}=(10^{-4},1)$, $\alpha_{\min}=(10^{-4},1)$, $\beta=(10^{-4},10)$, $\mu_{\max}=(10^{-3},10^{2})$, $\mu_{\min}=(10^{-3},10^{2})$. For other parameters shown in Table E, we set the E-P encounter distance $b=0.12\mu m$ (7). And when the E-P spatial distance is larger than $0.5\mu m$, the gene may not be active state (8-11). Thus, we assume the half-value distance of transcription rates is $\varepsilon_{1/2}=0.25\mu m$, and thus the rates drop to the minimum around $0.5\mu m$. We set $\delta=0.01$. If δ is taken as a multiple of 0.01, other inferred parameters will also change in multiples. The Hill coefficient h measures cooperativity. In biology, the h usually changes from 2 to 5 (12, 13). We fix $h=3$ for the studies. We repeatedly solve the optimization problem 10^{3} times with random initial values. Table B shows the minimize cross entropy (minH) for each model. Indeed, it has been shown that a two-state model with variable α and μ shows smaller minH than other models. We use this model to infer gene expression dynamics and the inferred results are shown in Table C. Table D indicate that the changes in d_{G} (corresponding to different monomer lengths) only leads to the multiplicative changes of k_{NN}, but parameter Θ in the Maxwell-Boltzmann distribution does not significantly change. Thus, selecting an appropriate length of the monomers for the study is sufficient.

Table B. Cross entropy minimization for different models that involving long-range E-P regulatory.

**Model**	Variable α	Variable μ	Variable $\alpha+\mu$
minH	11.7397	11.8636	**11.6865**

Table C. Parameter values for the best fitting model.

**Parameter**	k_{NN}	k_{EP}	γ	$\alpha_{\max}$	$\alpha_{\min}$	β	$\mu_{\max}$	$\mu_{\min}$
**Best fit**	0.7758	0.0969	28.1763	0.0414	0.0023	0.0992	2.1782	0.2726

Table D. Parameter values for the best fitting parameter in different monomer lengths.

**Parameter**	k_{NN}	k_{EP}	γ	$\alpha_{\max}$	$\alpha_{\min}$	β	$\mu_{\max}$	$\mu_{\min}$
Best fit (**5kb**)	**0.7758**	0.0969	28.1763	0.0414	0.0023	0.0992	2.1782	0.2726
Best fit (**2kb**)	**1.9916**	0.0938	29.8623	0.0372	0.0023	0.0783	1.9994	0.2309

Best fit (**1kb**)	**3.9046**	0.0972	26.5356	0.0355	0.0025	0.0774	2.0331	0.2052

Table E. Fixed parameter values for the fitting model.

**Parameter**	**Description**	**Value**	**Ref.**
k_B	Boltzmann constant	$1.38\times10^{-23}JK^{-1}$	
T	Room temperature	$300K$	
D	Monomer diffusion coefficient	---	$D\gamma = k_B T$
h	Hill coefficient	3	(13)
$\varepsilon_{1/2}$	Half-value distance of burst rates	$0.25\mu m$	(11)
b	E-P encounter distance	$0.12\mu m$	(7)
b'	a large distance (gene is inactive)	$0.5\mu m$	(8-11)

C. Comparing experimental data with theoretical results

We first study the relationship between E-P genomic distance and E-P contact probability. Based on the Maxwell-Boltzmann distribution, the cumulative PDF is

$$F\left(\Theta\left(k_{\mathrm{NN}}, k_{\mathrm{EP}}, d_{\mathrm{G}}, \gamma\right), d_{\mathrm{S}}\right) = erf\left(\frac{d_{\mathrm{S}}}{\sqrt{2}\Theta}\right) - \sqrt{\frac{2}{\pi}}\Theta^{-1} d_{\mathrm{S}} \exp\left(-\frac{d_{\mathrm{S}}^2}{2\Theta^2}\right), \quad [15]$$

where $\Theta = \sqrt{D\gamma\left(k_{\mathrm{NN}}/d_{\mathrm{G}} + k_{\mathrm{EP}}\right)^{-1}}$ and $erf\left(\cdot\right)$ is the error function defined as $erf\left(z\right) = 2\pi^{-1/2}\int_0^z e^{-t^2}\mathrm{d}t$. Therefore, the E-P encounter probability is $F\left(\Theta\left(k_{\mathrm{NN}}, k_{\mathrm{EP}}, d_{\mathrm{G}}, \gamma\right), \varepsilon_{\mathrm{T}}\right)$.

Second, we can investigate the relationship between E-P contact probability and mean mRNA level (and CV) according to the steady-state probability distributions.

D. Discussing with Zuin's model

In the literature (14), Zuin *et al.* proposed a theoretical model to explain the experimental data and uncover a potential mechanism of how enhancer act from large genomic distances. Here, we take a small discussion with Zuin's model and results.

Zuin's model assumed that the E-P spatial distance has two states (far and close) and these states have accumulating effects and eliminating effects to transmit information to the promoter. Essentially, it is a ON-OFF model that the OFF to ON rate is a constant coupled to the E-P interaction information (E-P encounter probability). However, our model uses a continuously fluctuating E-P spatial distance to directly transmit information to regulate the rate of gene-state switching. The OFF to ON rate and the transcription initiation rate are functions of E-P spatial distance. Generally, in the case of fast chromatin dynamics, upstream-to-downstream regulation in our model is performed through the expectation of the E-P spatial distance, which can be equated, in a general way, to the contact probability in Zuin's model.

Now, we take a briefly discuss of the results obtained by our model and Zuin's model. For the cell lines C_k ($k = 1,\ldots,6$), we can obtain the experimental steady mRNA distribution $P_i^E\left(x\right)$

(measured by the smRNA-FISH images data), the $P_i^W(x)$ (obtained by our model) and the $P_i^Z(x)$ (obtained by Zuin's model). In terms of the results of model fitting, we want to see which fitting of experimental data $P_i^E(x)$ is better. We calculate the cumulative distribution function (CDF) of above distribution and get $F_i^E(x)$, $F_i^W(x)$ and $F_i^Z(x)$. We use the Kolmogorov-Smirnov (KS) distance, defined by

$$KS_i^W = \max_x \left| F_i^W(x) - F_i^E(x) \right|,\ KS_i^Z = \max_x \left| F_i^Z(x) - F_i^E(x) \right|, \qquad [16]$$

to quantify the maximum distance between the distribution functions of two samples. S4A Fig shows the KS distance obtained by our model and by Zuin's model under different cell lines C_k. It can be seen that in only one of the six cell lines, the KS distance obtained by our fitting is larger than that of Zuin's model, and on the overall average, the KS distance obtained by our model is much smaller than that of Zuin's model. That is, to some extent, our model fitting results are better. S4B Fig shows the fitting results of the two models for cell line C_6, and S4C Fig shows the CDF and KS distance for cell line C_6.

Although more parameters in our model may fit the data better, we clarify the following points. First, fast chromatin dynamics only require the average E-P distance or connection probability, but slow dynamics need richer information and more parameters of the upstream chromatin motion. Second, we can infer the upstream and downstream parameters together using d_{G} data (that can be measured directly by experiments) rather than connection probability data (that needs to be computed) as well as gene expression distributions. Third, upstream parameters can effectively obtain the contact probabilities. To some extent, the number of downstream parameters is basically the same as Zuin's model. Finally, the results of the inference show that the model not only fits the data well but also infers the connection probabilities as a by-product, which reinforces the validity and reliability of the inference results.

We think that the coupling of the fast-slow chromatin dynamics would be an important factor contributing to the nonlinearity between transcriptional levels and E-P contact probabilities. In fact, Zuin's model also pointed out that nonlinearity might arise from the transient E-P interaction being translated to the slower transcription dynamics. However, we have carefully considered the different time scales between upstream and downstream based on real processes, provided a clear definition of scale gap between upstream and downstream, and elaborated on the role of fast and slow scales in gene expression regulation. In particularly, for the slow dynamics, the regulation of upstream to downstream is inseparable, which may cause the gene expression levels of the slow limit are not linear dependence on E-P contacts and eventually lead to the nonlinear behavior observed in the experiment.

E. Importance of slow chromatin dynamics

We find that the weight of progressively larger slow chromatin dynamics with decreasing d_{G} plays an important role in the modification of the fitted distribution (S5A Fig). For example, in cell

line C_2, we can show that the contribution of the slow dynamics is 32% and the coupled kinetics can adjust the non-original single peak in the fast case to an original single peak that is closer to the experimental data (S5C Fig). For clarity, we calculate the KS distances between fast distribution/coupled distribution and experimental, and then find that the average KS distance of the coupled distribution is smaller than that of the fast one (13% decrease on average and 33% decrease for cell cline C2, S5B Fig), showing that the slow chromatin dynamics play an important role in correcting the distribution. It should be noted, however, that the fact that these two KS distances are essentially the same for cell lines C_5 and C_6 does not mean that the slow kinetics has no contribution (the weights are 22% and 16% respectively), but the mRNA distributions produced by fast and slow dynamics are almost identical under the parameters (S5D Fig). In a word, the slow chromatin dynamics are crucial for the improvement of the data fitting.

Reference

1. K. Noma, C. D. Allis, S. I. Grewal, Transitions in distinct histone H3 methylation patterns at the heterochromatin domain boundaries. *Science* **293**, 1150-1155 (2001).
2. G. Thon, P. Bjerling, C. M. Bünner, J. Verhein-Hansen, Expression-state boundaries in the mating-type region of fission yeast. *Genetics* **161**, 611-622 (2002).
3. M. Bohn, D. W. Heermann, R. Van Driel, Random loop model for long polymers. *Phys. Rev. E* **76**, 051805 (2007).
4. C. W. Gardiner, *Handbook of stochastic methods for physics, chemistry and the natural sciences*. (Springer, 2004).
5. M. Yokoshi, K. Segawa, T. Fukaya, Visualizing the role of boundary elements in enhancer-promoter communication. *Mol. Cell* **78**, 224-235 (2020).
6. T. Fukaya, B. Lim, M. Levine, Enhancer control of transcriptional bursting. *Cell* **166**, 358-368 (2016).
7. D. I. Cattoni, A. M. C. Gizzi, M. Georgieva, M. Di Stefano, A. Valeri, D. Chamousset, C. Houbron, S. Déjardin, J.-B. Fiche, I. González, Single-cell absolute contact probability detection reveals chromosomes are organized by multiple low-frequency yet specific interactions. *Nat. Commun.* **8**, 1753 (2017).
8. J. M. Alexander, J. Guan, B. Li, L. Maliskova, M. Song, Y. Shen, B. Huang, S. Lomvardas, O. D. Weiner, Live-cell imaging reveals enhancer-dependent Sox2 transcription in the absence of enhancer proximity. *eLife* **8**, e41769 (2019).
9. L. Barinov, S. Ryabichko, W. Bialek, T. Gregor, Transcription-dependent spatial organization of a gene locus. arXiv preprint (2020).
10. T. Heist, T. Fukaya, M. Levine, Large distances separate coregulated genes in living Drosophila embryos. *Proc. Natl. Acad. Sci. U.S.A.* **116**, 15062-15067 (2019).
11. H. Chen, M. Levo, L. Barinov, M. Fujioka, J. B. Jaynes, T. Gregor, Dynamic interplay between enhancer–promoter topology and gene activity. *Nat. Genet.* **50**, 1296-1303 (2018).
12. J. N. Weiss, The Hill equation revisited: Uses and misuses. *FASEB J.* **11**, 835-841 (1997).
13. S. Bottani, R. A. Veitia, Hill function - based models of transcriptional switches: Impact of specific, nonspecific, functional and nonfunctional binding. *Biol. Rev.* **92**, 953-963 (2017).
14. J. Zuin, G. Roth, Y. Zhan, J. Cramard, J. Redolfi, E. Piskadlo, P. Mach, M. Kryzhanovska, G. Tihanyi, H. Kohler, Nonlinear control of transcription through enhancer-promoter interactions. *Nature* **604**, 571-577 (2022).
