# Supplementary figures and images for: 4D nucleome equation predicts gene expression controlled by long-range enhancer-promoter interaction
Source: PLoS Comput Biol. 2023 Dec 18;19(12):e1011722. doi: 10.1371/journal.pcbi.1011722 (PMC10760824; doi:10.1371/journal.pcbi.1011722)

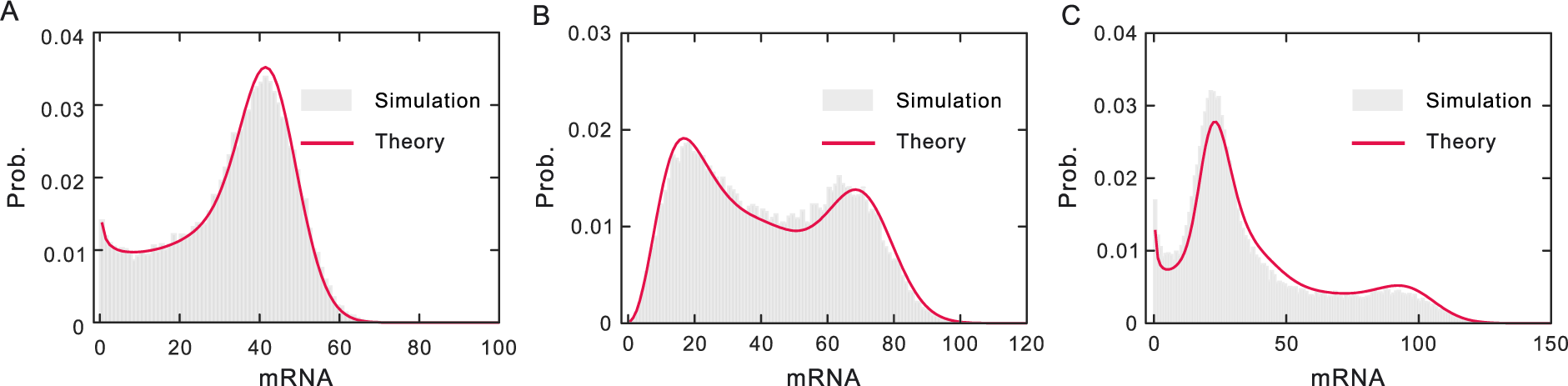

Supplement: S1 Fig — (A) The bimodal distribution (with a origin peak and a non-origin peak) of gene expression where kEP = 0.5, γ = 1, αmax = 0.10, αmin = 0.01, β = 0.03, μmax = 5, μmin = 2, δ = 0.1. (B) The bimodal distribution (with two origin peaks) of gene expression where kEP = 0.20, γ = 100, αmax = 5, αmin = 0.30, β = 0.50, μmax = 8, μmin = 2, δ = 0.1. (C) The trimodal distribution of gene expression where kEP = 0.1, γ = 300, αmax = 0.5, αmin = 0.05, β = 0.02, μmax = 10, μmin = 2, δ = 0.1. (TIF) [file pcbi.1011722.s002.tif]

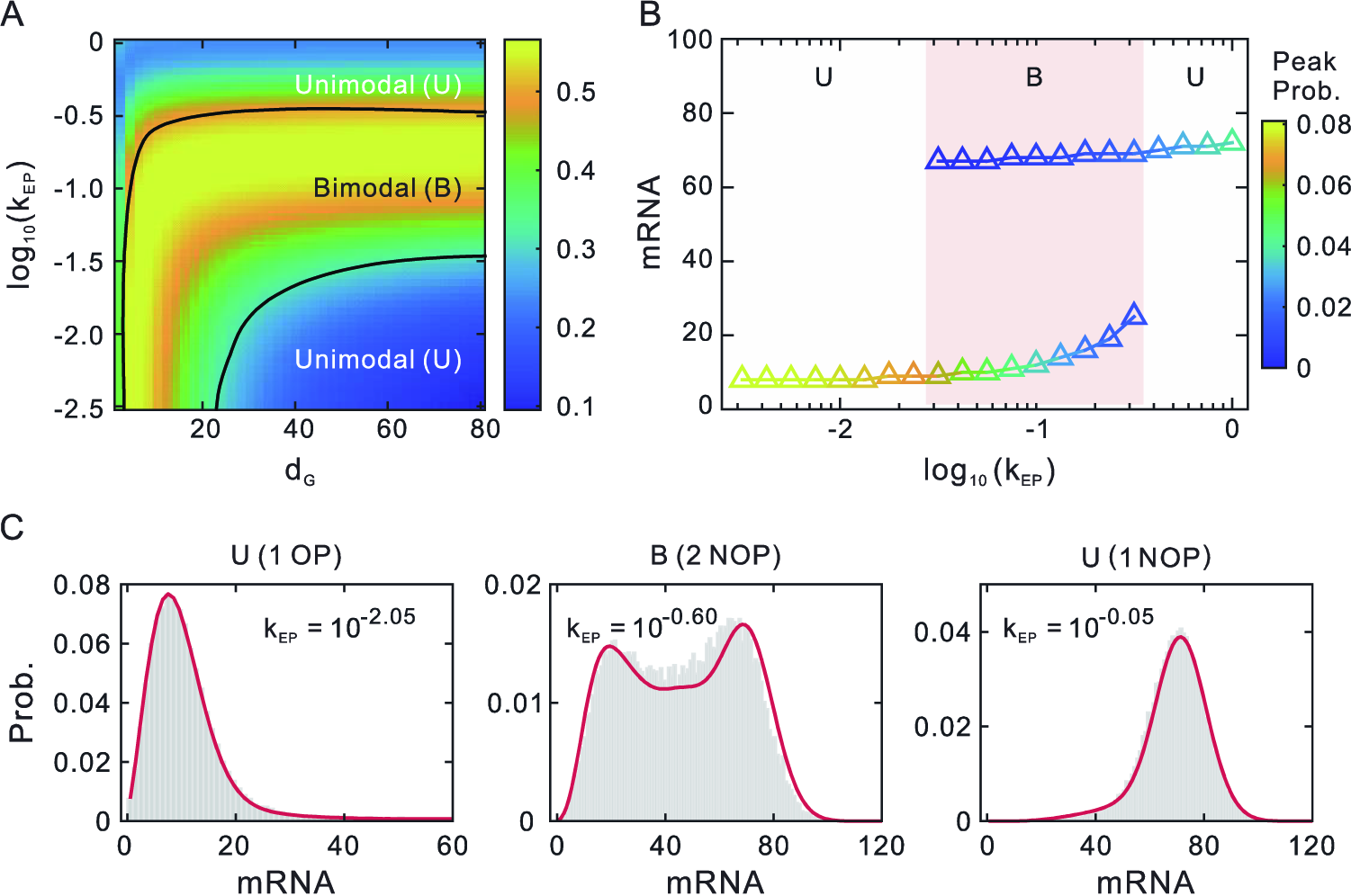

Supplement: S2 Fig — (A) Effects of E-P interaction on mRNA distribution. The black lines stand for the boundaries of different peak numbers. The plain is divided into distinctive regions representing unimodal (U) and bimodal (B). The heatmap is the bimodal coefficient. (B) Dependence of the most probable mRNA numbers on kEP where dG = 60. The color regions represent different peak numbers. The color bar represents the peak probabilities. (C) The example of unimodal/bimodal mRNA distribution. NOP (non-origin peak). The solid lines represent theoretical results and histograms represent numerical results. Parameter values are set as dG = 60, αmax = 5, αmin = 0.3, β = 0.5, μmax = 8, μmin = 2, δ = 0.1, γ = 50. (TIF) [file pcbi.1011722.s003.tif]

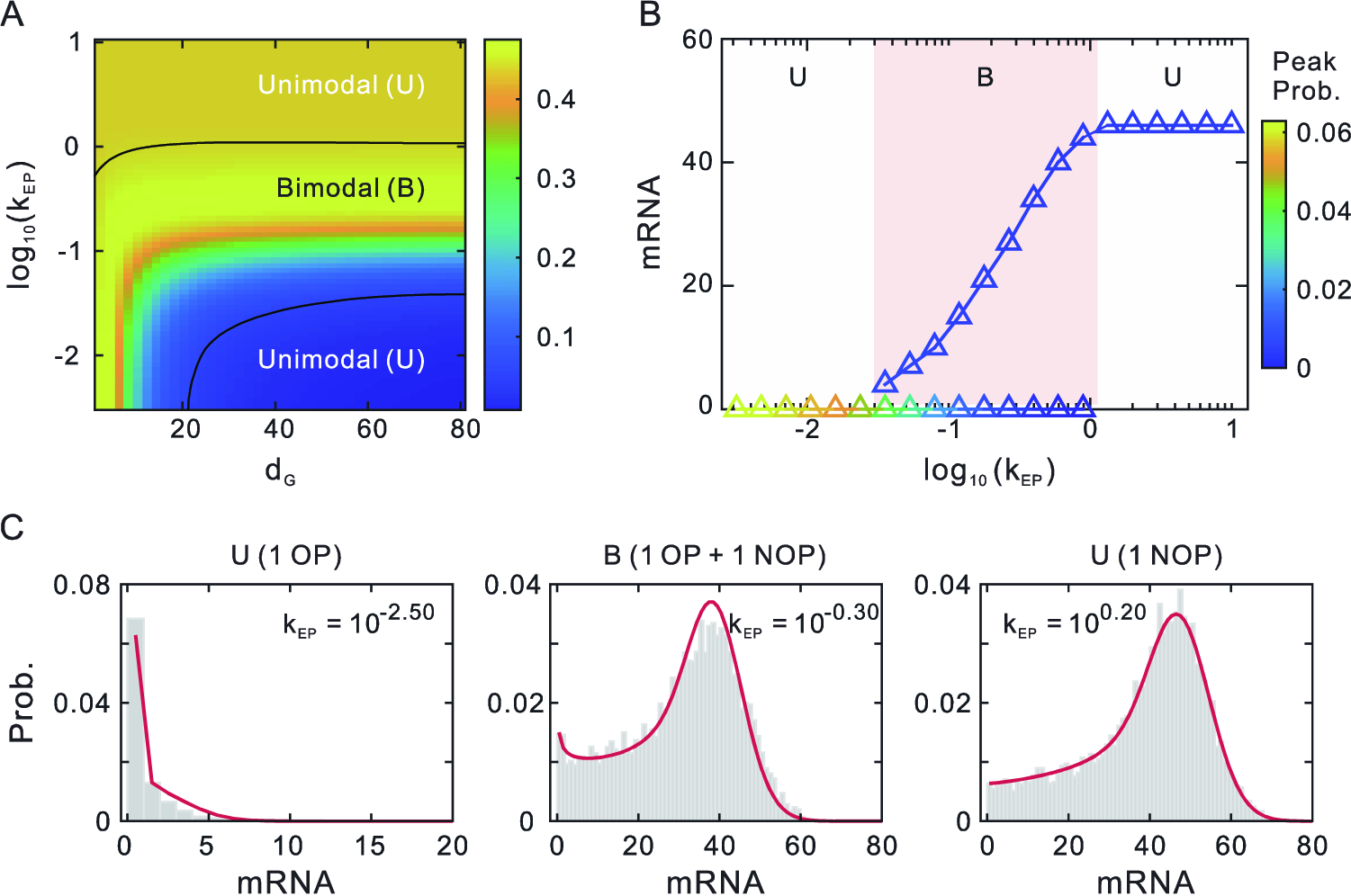

Supplement: S3 Fig — (A) Effects of E-P interaction on mRNA distribution. The black lines stand for the boundaries of different peak numbers. The plain is divided into distinctive regions representing unimodal (U) and bimodal (B). The heatmap is the bimodal coefficient. (B) Dependence of the most probable mRNA numbers on kEP where dG = 60. The color regions represent different peak numbers. The color bar represents the peak probabilities. (C) The example of unimodal/bimodal mRNA distribution. OP (origin peak), NOP (non-origin peak). The solid lines represent theoretical results and histograms represent numerical results. Parameter values are set as dG = 60, αmax = 0.10, αmin = 0.01, β = 0.03, μmax = 5, μmin = 2, δ = 0.1, γ = 1. (TIF) [file pcbi.1011722.s004.tif]

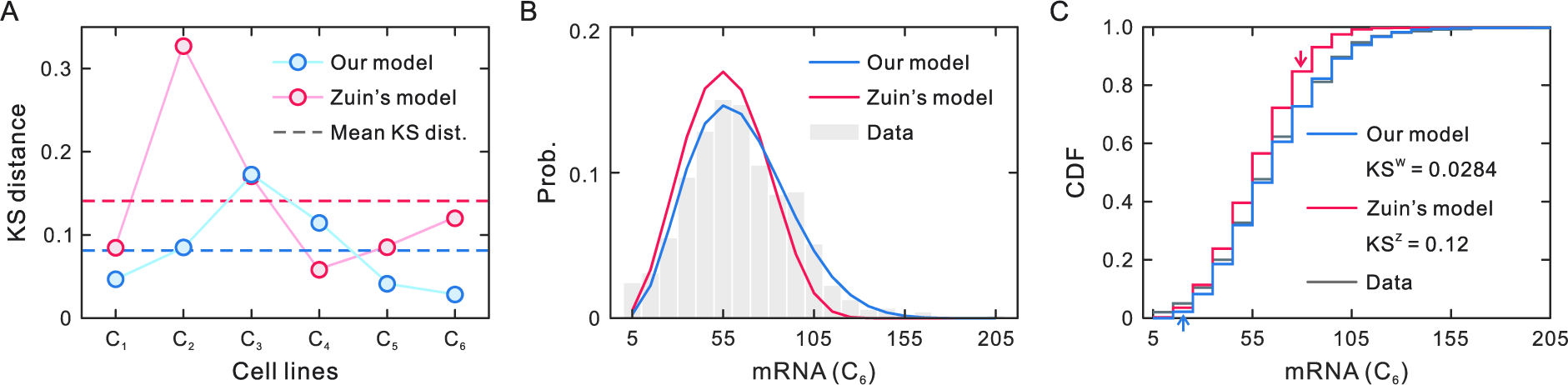

Supplement: S4 Fig — (A) KS distances of different cell lines (the KS distance is defined in Eq [16] in the S1 Text). The blue dots and shadow line stand for the KS distance between our model and experimental data, and the dashed blue line represents the mean KS distance for all cell lines. The red shows the KS distance between Zuin’s model and experimental data. (B) Distribution of mRNA numbers of smRNA-FISH in cell lines C6. The blue line shows the best fit of our model whereas the red line shows the best fit of Zuin’s model to the experimental data shown in the histograms. (C) CDF of the distributions in (B). The KS6W=0.0284 and KS6Z=0.12. The small arrows represent the number of mRNAs corresponding to the KS distance. (TIF) [file pcbi.1011722.s005.tif]

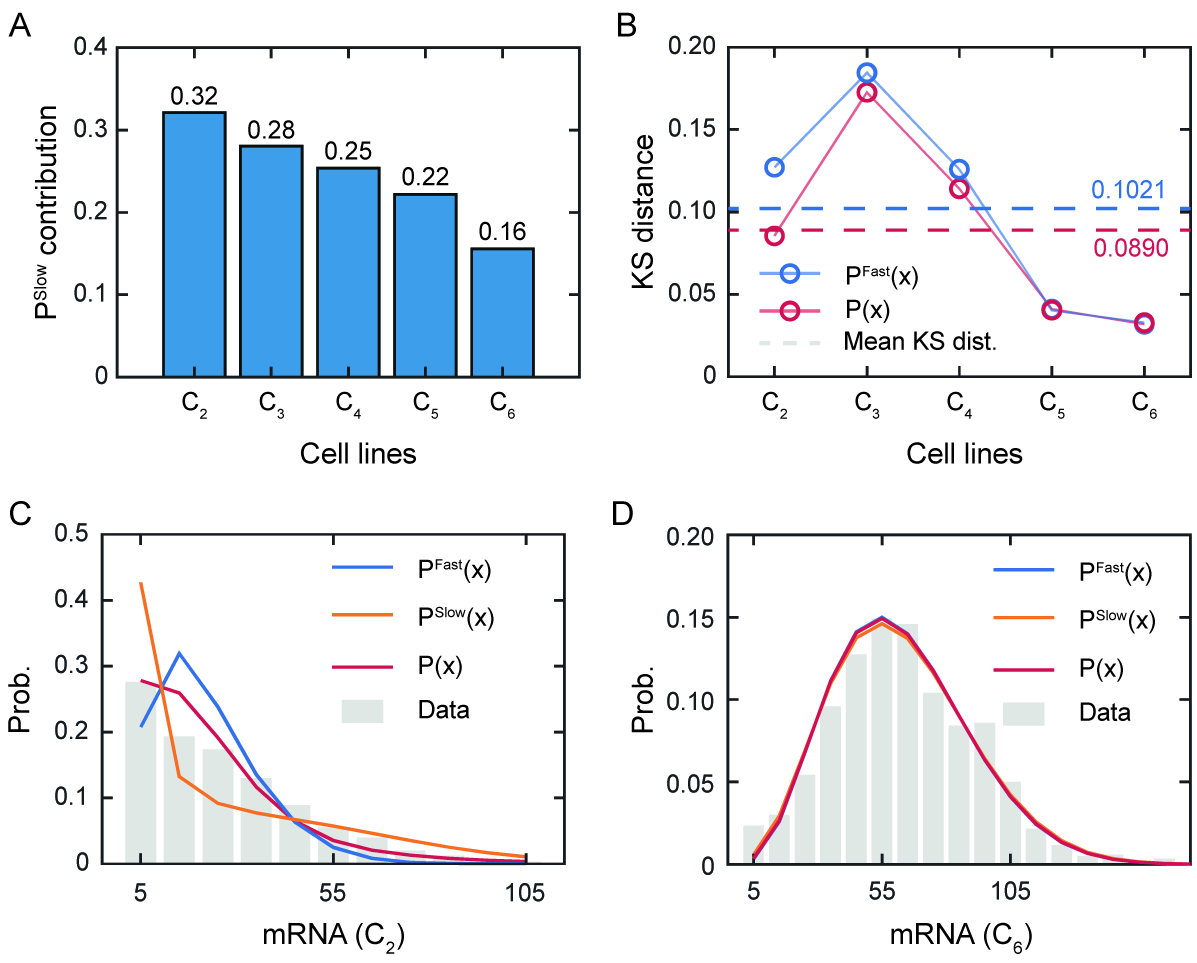

Supplement: S5 Fig — (A) The weight of slow chromatin dynamics in fitting distribution (1/(1+ω)). (B) KS distances of different cell lines. The blue dots and shadow line stand for the KS distance between the fast distribution and experimental data, and the red shows the KS distance between interpolation distribution and experimental data. The dashed lines represent the mean KS distance for all cell lines. (C) The distribution of Cell line 2. (D) The distribution of Cell line 6. (TIF) [file pcbi.1011722.s006.tif]

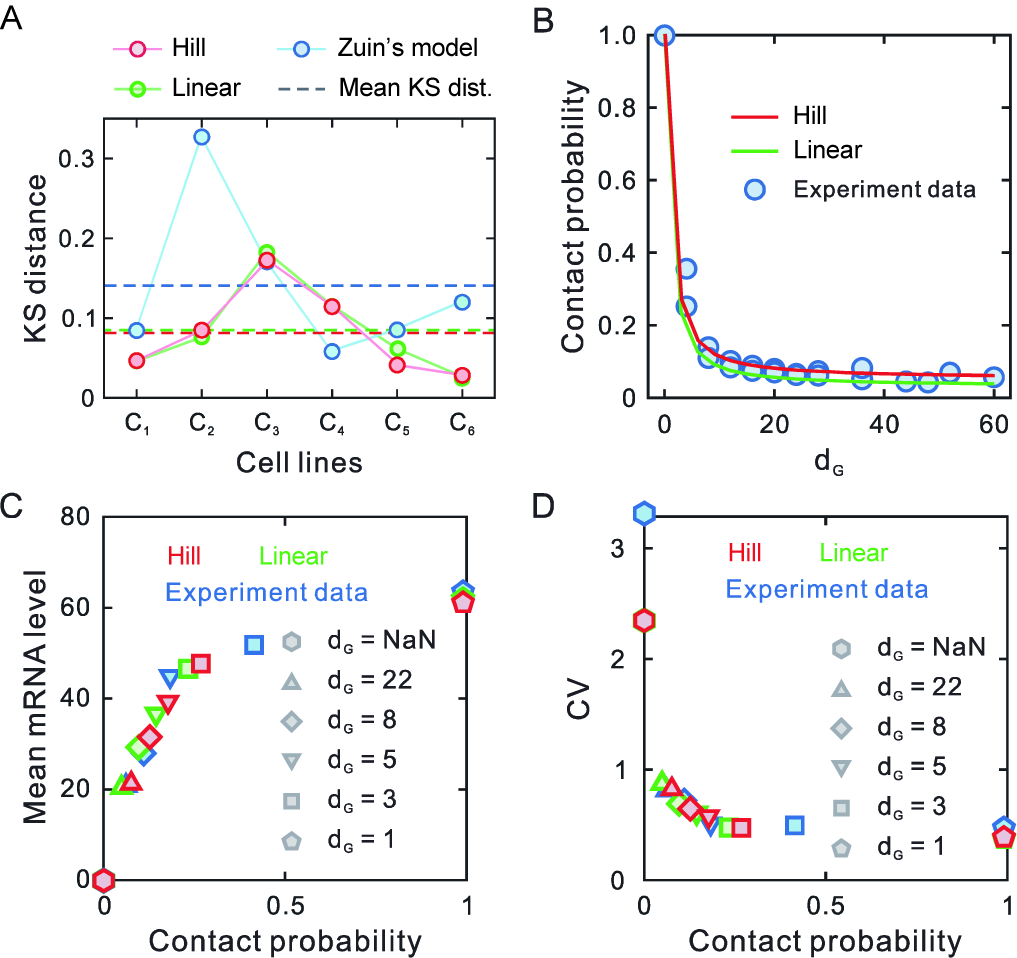

Supplement: S6 Fig — (A) KS distances of different cell lines. The red (/green) dots and shadow line stand for the KS distance between Hill (/linear) (Eq [3]/Eq [4] in S1 Text) dependence and experimental data. The blue shows the KS distance between Zuin’s model and experimental data. the dashed lines represent the mean KS distance for all cell lines. (B) The relationship between dG and contact probability. The blue circles represent the experimental data from the ectopic Sox 2 transgene. The solid red (/green) line is obtained based on the Hill (/linear) dependence. The inset shows the log-log plot. (C) Mean eGFP mRNA level plotted against contact probability between the ectopic Sox2 promoter and SCR insertions. The blue polygon shows the experimental data, and the red (/green) polygon shows the corresponding theoretical results based on the Hill (/linear) dependence. (D) CV of eGFP level against contact probability. The meanings of symbols are the same as those in (C). (TIF) [file pcbi.1011722.s007.tif]

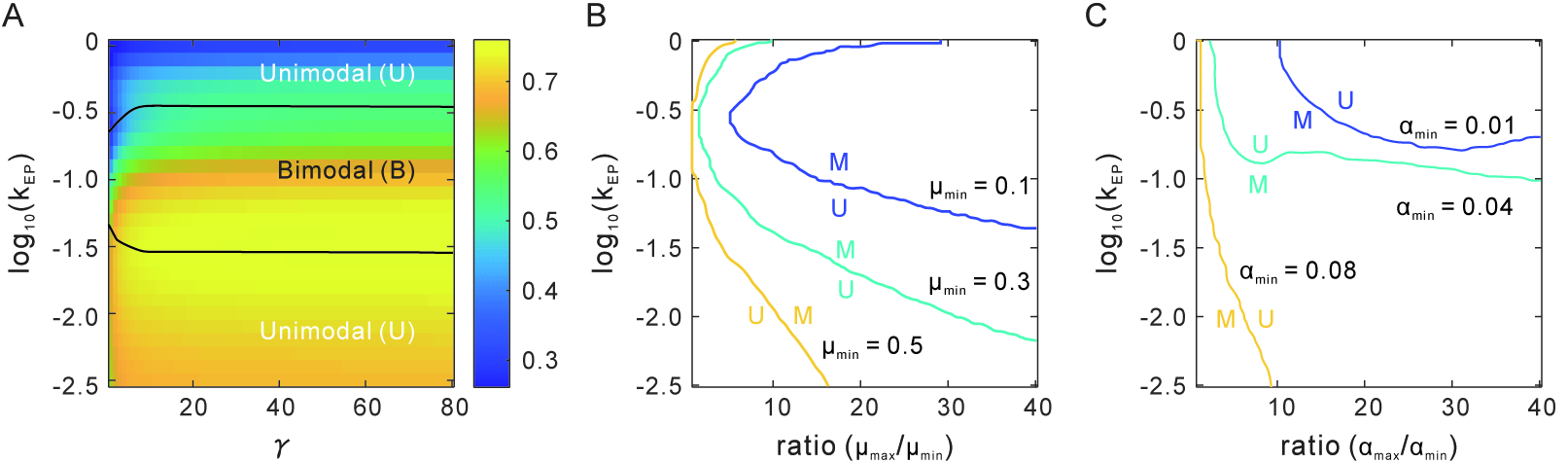

Supplement: S7 Fig — (A) Effects of E-P interaction strengths kEP and friction coefficient γ on the pattern of mRNA distribution. Parameters are dG = 60, αmax = 5, αmin = 0.3, β = 0.5, μmax = 8, μmin = 2, δ = 0.1. (B) Effects of kEP and the ratio of μmax/μmin on the pattern of mRNA distribution. The μmin = 0.1, 0.3, 0.5. Parameters are αmax = 0.10, αmin = 0.01. (C) Effects of kEP and the ratio of αmax/αmin on the pattern of mRNA distribution. The αmin = 0.01, 0.04, 0.08. Parameters are μmax = 5, μmin = 2. Each line divides the entire region into two parts, with U indicating a single peak area and M indicating a multimodal area. Other parameter values are set as dG = 60, β = 0.03, δ = 0.1, γ = 1. The solid line divides the entire region into two parts, with the part marked U indicating unimodal and the part marked M indicating multimodal (including bimodal and trimodal). (TIF) [file pcbi.1011722.s008.tif]
