# Supplementary material for: 4D nucleome equation predicts gene expression controlled by long-range enhancer-promoter interaction
Source: PLoS Comput Biol. 2023 Dec 18;19(12):e1011722. doi: 10.1371/journal.pcbi.1011722 (PMC10760824; doi:10.1371/journal.pcbi.1011722)
Supplement: Attachment — Submitted filename: ResponseLetter-PLoSCB.pdf [file pcbi.1011722.s009.pdf]

Response Letter

4D nucleome equation predicts gene expression controlled by long-range enhancer-promoter interaction [PCOMPBIOL-D-23-00602]

Zihao Wang[1,2], Songhao Luo[1,2], Zhenquan Zhang[1,2], Tianshou Zhou[1,2,*], and Jiajun Zhang[1,2,*]

[1] Guangdong Province Key Laboratory of Computational, Sun Yat-sen University, Guangzhou 510275, People's Republic of China

[2] School of Mathematics, Sun Yat-Sen University, Guangzhou 510275, People's Republic of China

Dear the Editors and Reviewers,

Thank you for your precious time and kind comments on this research and also thanks for providing this valuable opportunity to revise our manuscript. We extremely cherish this opportunity and try to do our best to make revisions. All the revisions we made have been indicated in red color to allow the reviewers to locate these parts easily. In order to fix the reviewers' concerns, we have also added or supplemented some text contents and figures in the Supplementary File where we have given interpretations on issues about what the reviewers concerned. In a word, we have answered all the questions raised in the three review reports point by point, and would believe that reviewers are satisfied with our revisions.

Best regards!

Tianshou Zhou and Jiajun Zhang

On behalf of Zihao Wang, Songhao Luo, Zhenquan Zhang

School of Mathematics

Sun Yat-sen University

P.R.C. China

Email: mcszhtsh@mail.sysu.edu.cn, zhjiajun@mail.sysu.edu.cn

Reviewer #1:

In the manuscript '4D nucleosome equation predicts gene expression controlled by long-range enhancer promoter interaction' Wang et al. proposed a theoretical framework for studying gene expression dynamics as a result of enhancer promoter (E-P) interaction. The fundamental concept of the framework is the description of chromatin dynamics and gene expression dynamics in a single equation, as well as the separation of the timescales for the two types of dynamics. The authors showed that simulations based on this framework produced results consistent with both the general understanding of E-P regulation and specific experimental data. Overall, the modeling framework is rigorous and novel. The manuscript is well-written overall. I have a few comments/suggestions regarding the connection between the models and data, as well as the clarity of the manuscript.

Response: Thanks for your positive evaluation of our study. In what follows, we address all the issues raised in your review report by point-to-point. We hope you are satisfied with the revised version.

1. The biological interpretation of the 'monomer' is unclear. It was introduced generically on Page 5 without a description of its meaning. On Page 7, however, it was indicated that each monomer is one nucleosome. When fitting the model to the mESC data, a monomer became a 5kb region, which is more than 10-fold longer than a region spanned by a nucleosome. The confusing usages of the monomer's meaning needs to be addressed. While the authors can make the interpretation flexible, doing so would mean that the biological meanings for all parameters of the model will change accordingly. This can be problematic when interpreting the simulation results. In addition, it will be helpful if the authors can use realistic distributions of genomic E-P distances to explain the scales of their structural model.

Response: Thanks for your comment. To model a chromatin region, most work has used a much lower level of detail whereby a chromatin fiber is represented by a connected chain of monomers. **Each monomer represents a segment of DNA**, whose length depends on the levels of coarse-grained chromatin or depends on the question being asked. During the fitting of mESC data, **different monomer lengths do not change the biological meanings of the parameters or the fitting results.** The Additional Table **1** shows the best-fit parameters using different monomer lengths (5kb, 2kb,1kb), corresponding to different d_G. We find that the change in d_G only leads to the elasticity coefficient k_NN multiplicative changes, which results in basically unchanged of k_NN/d_G (or the parameter in the Maxwell-Boltzmann distribution). For example, for cell line C2, the values of the d_G for different monomer lengths are 22 (5kb), 56 (2kb) and 112 (1kb). Therefore,

the ratio k_NN/d_G is 0.0353 (=0.7758/22), 0.0356 (=1.9916/56) and 0.0349 (=3.9046/112), three being approximately equal. From a physical viewpoint, this can be explained as a coarsening process. That is, the elasticity coefficient k_NN in the 5kb model can be viewed as the equivalent spring coefficient of 5 springs in series in the 1kb system.

The distribution of E-P genomic distances is generally in an unimodal form, and as the d_G increases, the probability distribution first increases and then decreases. In mouse, the median genomic distances are about 280 kb and the most possible distances are about 150 kb [1]. For example, if one monomer represents 5kb regions, then 30~60 monomers are required to simulate the E-P genomic distance. And if one monomer represents 2kb regions, then 70~150 monomers are required. Importantly, based on the above results, the length of the monomers does not affect the final theoretical and inferred results, so selecting an appropriate length of the monomers for the study is sufficient to study the gene expression controlled by the E-P interaction.

Additional Table 1. Parameter values for the best fitting parameter in different monomer lengths.

Parameter	k_{NN}	k_{EP}	γ	α_{max}	α_{min}	β	μ_{max}	μ_{min}
Best fit (**5kb**)	**0.7758**	0.0969	28.1763	0.0414	0.0023	0.0992	2.1782	0.2726
Best fit (**2kb**)	**1.9916**	0.0938	29.8623	0.0372	0.0023	0.0783	1.9994	0.2309
Best fit (**1kb**)	**3.9046**	0.0972	26.5356	0.0355	0.0025	0.0774	2.0331	0.2052

[1] Yu, Miao, et al. Integrative analysis of the 3D genome and epigenome in mouse embryonic tissues. *bioRxiv* (2022): 2022-04.

2. The comparison of multimodality of mRNA distribution from simulations and experimental data seems weak. Out of the three references mentioned in that section on Page 16, none of them showed the relationship between E-P regulation and modality of mRNA distribution. It is therefore unclear whether the simulation results are realistic.

Response: Thanks for your comment. Gene expression is regulated by E-P communication, so the regulatory information from E-P communication is already contained within the experimentally observed mRNA distribution. In Ref [2], the researchers studied the transcriptional behavior of GREB1 changes with estrogen dose. Generally, addition of estrogen (17b-estradiol, E2) may increase contacts between the GREB1 gene and the estrogen-receptor-alpha-bound enhancer [3], thus altering the E-P interaction strength in our model. When increasing the E2 concentration (increasing the E-P interaction strength), we find that the mRNA level grows, and the mRNA distribution changes from the single peak to a bimodal distribution. **The changing behavior is consistent with what we described in the paper.** A new peak emerges at a larger mRNA level

and gradually increases and the peak near the origin gradually decreases. In addition, we have cited other related papers to show that our model can produce the experimentally observed bimodal phenomenon. To make the description more concise and accurate, we have modified the corresponding parts of the main text.

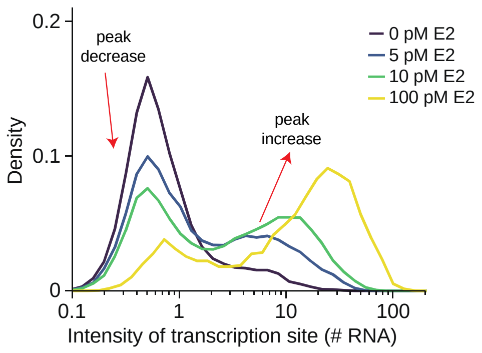

Additional Figure 1. Increasing E2 increases the proportion and productivity of transcriptional (from Ref. [2]).

[2] Fritzsch, C., et al., Estrogen-dependent control and cell-to-cell variability of transcriptional bursting. *Mol. Syst. Biol.*, 2018, **14**(2), e7678.

[3] Hevener, A. L., Ribas, V., Moore, T. M., & Zhou, Z., ERα in the control of mitochondrial function and metabolic health. *Trends Mol. Med.*, 2021, **27**(1), 31-46.

3. I suggest that the authors use their framework to explain the source of the multimodality. Is this a result of the chromatin dynamics which produced multiple structural configurations that are more stable than others? If yes, some analysis of the structural model component will be helpful.

Response: Good question! Only the downstream ON-OFF model could **not** produce bimodality with a non-origin peak, even less likely trimodality. Our model of gene transcription has the potential to produce multimodality. We think that the **regulation of chromatin dynamics, especially E-P regulation, is a main source of multimodality** in gene expression distribution. In our model, we used **a unimodal** E-P stationary spatial distance (Maxwell-Boltzmann distribution) to modulate switching between gene states.

However, whether chromatin dynamics produce multiple stable structural configurations and thus lead to multimodal gene expression distributions is a good question. For this, we do not have a clear answer. Our model represents that a single stable structural configuration can lead to bimodal and trimodal. This is mainly due to **differences in timescales between upstream and downstream**. It seems that the multiple structural configurations are likely to lead to a multimodal gene expression distribution. We need a deeper investigation of this question in future research.

4. Page 3, Line 56. ‘Its’ should be ‘whose’, and ‘chromatin’ is misspelled.

Response: We have corrected this.

5. Page 14, the last sentence. ‘Decrease’ should be ‘decreases’, and ‘trends’ should be ‘tends’.

Response: Thanks. This has been corrected.

Reviewer #2

The paper introduces the 4D nucleome equation as a comprehensive theoretical framework for investigating the impact of long-range enhancer-promoter (E-P) interactions on gene expression dynamics. The framework incorporates upstream chromatin motion, downstream mRNA production, and the temporal connection between these processes. The authors derive analytical mRNA distribution and explore how E-P interactions qualitatively influence the characteristics of mRNA distribution. The 4D nucleome equation provides a valuable modeling framework for studying the influence of chromatin dynamics on gene expression kinetics.

Response: We are pleased that the referee appreciates our work.

However, it is unclear what specific contributions the authors have made compared to previous work. Although the authors briefly discuss a comparison with the model presented in Ref. 10, most of the discussion is relegated to the Supplementary Information (SI). It appears that the authors' model is identical to the one in Ref. 10 in the fast chromatin dynamics regime. In this limit, the contact probability used in Ref. 10 can be directly replaced with the distance used in Eq. 3 of the SI.

Response: Thanks for your comment. We compare to previous work and emphasize our specific five contributions as follows.

First, we developed **a general theoretical framework to study gene expression regulated by E-P communication**, which transforms a complex biological issue into a mathematically solvable one. Gene expression is regulated by spatial chromosome topology and previous models commonly ignored this process. Meanwhile, we would point out that the random organization of chromatin can cause fluctuations in E-P spatial distance and regulate gene transcription **in real-time** [1]. Therefore, time-varying state-switching rates between promoter states may be a more realistic and practical modeling approach to promoter state switching regulated by E-P interaction with cumulative effects (multi-steps) described in Ref. 10.

Second, we obtained **analytical results for the E-P spatial distance distribution and mRNA distributions regulated by E-P communication**. We explicitly derived the E-P spatial distance distribution as Maxwell-Boltzmann (MB) distribution and the data in living mouse embryonic stem cells have indicated the validity. The characteristic parameter of MB distribution clearly defines an analytical relationship between E-P spatial distance and E-P interaction parameters (including genomic distance and interaction strength). Meanwhile, we obtain a high-accuracy approximation of mRNA steady distribution which is a useful and simple formula for predicting the dynamics of mRNA over a broad range of timescale separation.

Third, our model demonstrated that **E-P communication is a flexible regulation strategy to explain possible implications of bimodal distributions and even trimodal distributions.** It is important to point out that without the contribution of slow chromatin dynamics, the obtained Poisson-Beta distribution of fast dynamics can produce neither the bimodal with two non-origin peaks (observed in the experiment [2]) nor the trimodal, implying **the importance of slow chromatin dynamics in regulating gene-product distributions** (as pointed out in the reviewer's next comment). This provides insights into complex mechanisms of biological processes and is essential for cellular decision-making and environmental adaptation. Note that in spite of the complexity of the expression of the switching rate in Ref. 10, it is essentially a constant and the mRNA distribution is a Poisson-Beta distribution. So, **it is impossible that the bimodal with two non-origin peaks or the trimodal appears in the model in Ref. 10**.

Fourth, we can **use less information or directly measured data to infer gene expression dynamics regulated by E-P interactions, and this will have wide applications.** In fact, only the E-P genomic distance and the gene expression distribution are needed for inference, without needing contact probability data which are more difficult to measure than the E-P genome distance. Therefore, our model has a wider range of applications than Ref. 10.

Fifth, our model can **illustrate the nonlinear relationship observed in the experiment between transcriptional levels and E-P contact probabilities**. We find that the Hill or linear upstream-to-downstream connection can effectively fit the experimental data. Therefore, the nonlinear dependence of the activation rate on E-P contacts does not seem to be a dominant cause of the nonlinear relationship observed in the experiment (as pointed out in the reviewer's comment). Rather, **the coupling of the fast-slow chromatin dynamics may be an important factor contributing to the nonlinearity**. And in particularly, for the slow chromatin dynamics, the regulation of upstream to downstream is inseparable, which may cause the gene expression levels of the slow limit are not linear dependence on E-P contacts. In addition, the Ref. 10 also pointed out that nonlinearity might arise from the transient E-P interaction being translated to the slower transcription dynamics. However, we have carefully considered the different time scales between upstream and downstream based on real processes, provided a clear definition of scale gap between upstream and downstream, and elaborated on the role of fast and slow timescales in gene expression regulation.

Finally, we point out that in the case of fast chromatin dynamics, upstream-to-downstream regulation is performed through the expectation of the E-P spatial distance, which can be equated, in a general way, to the contact probability in Ref. 10. However, the slow chromatin dynamics also plays an important role in regulating the distribution of mRNAs and we derive the corresponding distribution for this case (as pointed out in the reviewer's next comment). The general regulation of

gene expression by E-P communication should be the coupling of fast and slow scales, and we derive an approximate formula for mRNA distribution. In this revision, we have discussed the model presented in Ref. 10 in the main text Discussion part.

[1] Chen, H., et al., Dynamic interplay between enhancer–promoter topology and gene activity. *Nat. Genet.*, 2018, **50**(9), 1296-1303.

[2] Fritzsch, C., et al., Estrogen-dependent control and cell-to-cell variability of transcriptional bursting. *Mol. Syst. Biol.*, 2018, **14**(2), e7678.

Additionally, the authors introduce expressions for the slow chromatin dynamics limit, which are new. However, they do not discuss the contribution of slow dynamics to the fitting of experimental data. While the authors emphasize that their model fits experimental data better than the expression in Ref. 10, this improvement may be expected since the new model has more parameters. It would be interesting if the authors could provide insight into the obtained value for w in Eq. 12 and its impact on fitting with experimental data. Is the contribution from slow chromatin dynamics important for the observed improvement in fitting?

Response: Thanks for your suggestion. First, the basic fit results allow us to calculate the weight (or contribution) of the slow-scale dynamics to the data fit ($1/(1+\omega)$). We find that the weight of the progressively larger slow chromatin dynamics as d_G decreases (Additional Figure **2A**) plays an important role in the modification of the fitted distribution. For example, in cell line C2, we can see that the contribution of the slow dynamics is 32% and the coupled kinetics can adjust the non-original single peak under the fast to an original single peak that is closer to the experimental data (Additional Figure **2C**). Second, we calculate the KS distances between the fast distribution (or coupled distribution) and experimental distribution, finding that the average KS distance of the coupled distribution is smaller than that of the fast one (13% decrease on average and 33% decrease for cell cline C2, Additional Figure **2B**), which also illustrates the role of the slow chromatin dynamics in correcting the distribution. It should be noted, however, that the fact that these two KS distances are essentially the same for cell lines C5 and C6 does not mean that the slow kinetics has no contribution (the weights are 22% and 16% respectively), but rather that the distributions produced by fast and slow are almost identical under the same parameters (Additional Figure **2D**). In a word, the slow chromatin dynamics are crucial for the improvement of the data fitting. We have explicitly emphasized the importance of the contribution of slow dynamics in the main text.

Although more parameters may fit the data better, we clarify the following points. **First**, fast chromatin dynamics only require the average E-P distance or connection probability, but slow

dynamics need richer information and more parameters of the upstream chromatin motion. **Second**, we can infer the upstream and downstream parameters together using d_G data (that can be measured directly by experiments) rather than connection probability data (that needs to be computed) as well as gene expression distributions. **Third**, upstream parameters can effectively obtain the contact probabilities. To some extent, the number of downstream parameters is basically the same as the model in Ref. 10. **Finally**, the results of the inference show that the model not only fits the data well but also infers the connection probabilities as a by-product, which reinforces the validity and reliability of the inference results.

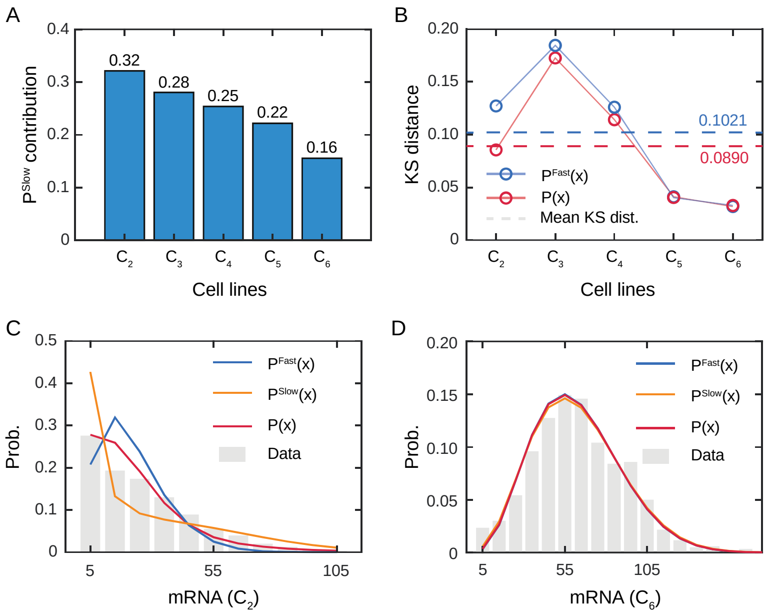

Additional Figure 2. The contribution of slow chromatin dynamics to data fitting. (A) The weight of slow chromatin dynamics in fitting distribution with factor $1/(1+\omega)$. (B) KS distances of different cell lines. The blue dots and shadow line stand for the KS distance between the fast distribution and experimental data, and the red shows the KS distance between interpolation distribution and experimental data. The dashed lines represent the mean KS distance for all cell lines. (C) The distribution of Cell line 2. (D) The distribution of Cell line 6.

To justify the publication of the manuscript, the authors need to highlight novel observations that were not reported in Ref. 10. Many of the analytical expressions presented in the manuscript are standard for polymer physics and stochastic gene expression models. While it is commendable that the authors report these expressions and combine the two models, the expressions alone may not present sufficiently interesting results for publication.

Response: Thanks for your comment. In addition to the general theoretical framework established to study gene expression regulated by E-P communication and the theoretical results obtained

regarding the E-P spatial distance distribution and mRNA distributions, we emphasize our novel research and observational results that were not reported in Ref. 10 as follows.

First, the regulation of gene expression by E-P interaction can generate bimodal distributions and even trimodal distributions. It is impossible that the bimodal with two non-origin peaks or the trimodal appears in Ref. 10's model.

Second, slow chromatin dynamics and its limit, which are the special aspect compared to Ref.10, plays an important role in regulating gene-product distributions.

Third, the coupling of the fast-slow chromatin dynamics rather than the nonlinear dependence of the activation rate on E-P contacts could be an important factor contributing to the nonlinearity observed in the experiment between transcriptional levels and E-P contact probabilities.

Fourth, compared to Ref. 10, we can use less information or directly measured data to infer gene expression dynamics regulated by E-P interactions (without needing contact probability data), and this will have wide applications.

The first three key points reveal the importance of complex upstream and downstream regulation on gene expression distributions and the nonlinear relationship between E-P contact probabilities and gene expression levels. The last point is for the wider application of our model. We have explicitly elaborated these novel observations in the main text.

Furthermore, one of the most notable findings in Ref. 10 is the non-linear dependence of the activation rate on E-P contacts, as demonstrated in Eq. 3 of the SI. Surprisingly, the authors do not mention this non-linearity in the main text and simply adopt the same assumptions as in Ref. 10. This omission prevents the authors from providing alternative explanations for the underlying molecular mechanisms that give rise to the observed non-linearity.

Response: Thanks for your suggestion. In Ref. 10, the authors found that the transcriptional effect of an enhancer depends on its contact probabilities with the promoter through a nonlinear relationship in experimental data, and use a 'variable' two-state model with a non-linear activation rate to explain this phenomenon.

In our model, we first use a nonlinear Hill function to link the upstream and downstream. Here, we consider changing the upstream and downstream nonlinear Hill function in the model to a linear form (i.e., using a linear function within a reasonable range of E-P spatial distances). By using the same fitting method, we then find that the distributions fitted by the linear model are overall better than those fitted by Zuin's model (see the following additional Figure **3A**). We also find that the linear model fits the relationship between d_G and contact probability well, and predicted gene

expression levels and CVs well (Additional Figure **3B-D**). Therefore, for our model, non-linear dependence of the activation rate on E-P contacts **seems to be not a dominant cause** of the nonlinear relationship between connection probability and mean expression observed in the experiment.

Rather, we think that **the coupling of the fast-slow chromatin dynamics would be an important factor contributing to the nonlinearity**. In fact, the Ref. 10 also pointed out that nonlinearity might arise from the transient E-P interaction being translated to the slower transcription dynamics. However, we have carefully considered the different time scales between upstream and downstream based on real processes, provided a clear definition of scale gap between upstream and downstream, and elaborated on the role of fast and slow scales in gene expression regulation. In particularly, for the slow dynamics, the regulation of upstream to downstream is inseparable, which may cause the gene expression levels of the slow limit are not linear dependence on E-P contacts and eventually lead to the nonlinear behavior observed in the experiment. In the main text and Supplementary Text, we have added detailed discussions about this nonlinearity.

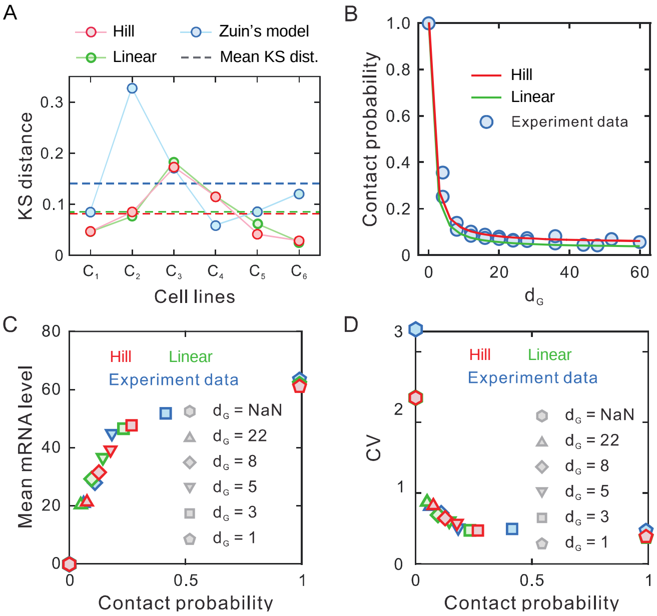

Additional Figure 3. Comparisons between the fitting results of model's Hill (non-linearity) dependence and linear dependence to experimental data. (A) KS distances of different cell lines. The red (/green) dots and shadow line stand for the KS distance between Hill (/linear) dependence and experimental data. The blue shows the KS distance between Zuin's model and experimental data. the dashed lines represent the mean KS distance for all cell lines. (B) The relationship between d_G and contact probability. The blue circles represent the experimental data from the ectopic Sox2 transgene. The solid red (/green) line is obtained based on the Hill (/linear) dependence. The inset shows the log-log plot. (C) Mean eGFP mRNA level plotted against contact probability between the ectopic Sox2 promoter and SCR insertions. The blue polygon shows the

experimental data, and the red (/green) polygon shows the corresponding theoretical results based on the Hill (/linear) dependence. (D) CV of eGFP level against contact probability. The meanings of symbols are the same as those in (C).

Lastly, there are some minor comments that the authors should address.

1) Firstly, if Eq. 9 in the main text has been derived previously, the authors should provide appropriate citations or clarify the novelty of the expression.

Response: We thank you for pointing out this point. We have provided appropriate citations.

2) Secondly, for the fitting presented in Fig. 5, it would be useful to list the parameters in the SI.

Response: Thank you for your good suggestion. We have listed the parameters in the SI.

3) Lastly, for Fig. S4, the authors should refer to the SI text that provides the definition of the KS distances.

Addressing these points will strengthen the manuscript and enhance its potential for publication.

Response: We thank you for pointing out this point. We have added a reference of the definition of KS distances in Fig. S4.

Reviewer #3

The authors developed a modeling framework to couple enhancer-promoter interaction with gene expression at different timescales and applied the model to study how the enhancer-promoter interaction affects gene expression dynamics. It considered upstream chromatin motion on a fast timescale and downstream mRNA production on a slow timescale. The authors derived analytical mRNA distribution by following timescale separation method and also numerically solved the equation. They showed that E-P interaction could give rise to multiple shapes of mRNA distributions including bimodal and trimodal distributions. A power-law scaling of gene expression levels in the E-P genomic distance was also demonstrated through both theoretical analysis and numerical simulations. Analysis of experimental data showed consistent results as predicted by the model in mRNA distribution and E-P contact probability under different E-P interactions. The manuscript was clearly written to describe the modeling framework, main results and conclusions, except some grammar mistakes. I would suggest publishing this work if the following comments can be addressed:

Response: The reviewer has succinctly summarized the major findings of our work and we appreciate this summary. In what follows, we address the issues raised in the review report. We hope the referee is satisfied with the revised version.

On page 6, in the equation F(r,s;t)=V(r,s:t)-∇_r·(Dlogp(r;t)), it should be ∇_r (Dlogp(r;t)) instead of ∇_r·(Dlogp(r;t)).

Response: Thanks for your careful review. This has been corrected.

On page 8, please explain why overdamped Langevin equation was applied to model the chromatin dynamics in more detail.

Response: Since the aqueous environment (cytosol) of a cell is highly viscous at the length and velocity scales of macromolecules (low Reynolds number), the chromatin motion can be treated as an overdamped system where inertial effects are ignored. This prevents any particles (molecules or other diffusing bodies) from accelerating into any sort of ballistic motion. Many papers do not specifically mention the overdamping when using the Langevin equation [1, 2]. To make our work more concise and easier to read, we deleted this description.

[1] Shukron, O. and Holcman, D., Transient chromatin properties revealed by polymer models and stochastic simulations constructed from Chromosomal Capture data. *PLoS Comput. Biol.*, 2017, **13**(4), e1005469.

[2] Shukron, O., Piras, V., Noordermeer, D., & Holcman, D., Statistics of chromatin organization during cell differentiation revealed by heterogeneous cross-linked polymers. *Nat. Commun.*, 2019, *10*(1), 2626.

On page 10, when minimizing the total cross entropy function Eq. [6], was it possible to obtain multiple local minimizers? In that case, how were the parameter values determined? Will conclusions still be valid for different parameter values?

Response: Thanks for your comment. First, for the parameters to be determined, we will select different initial values for multiple simulations, and each simulation will yield a local minimum and a set of parameter values. Second, we will sort the results of each simulation according to the cross-entropy function values to obtain a global minimum. In fact, after sorting, we can obtain, from the results of multiple simulations, the global minimum with the same cross entropy and the same set of parameter values.

On page 13 line 265, V(r)=[(k_NN/d_G +k_EP) d_s]/bγ should be V(r)=[(k_NN/d_G +k_EP) d_s]/(bγ)?

Response: Thanks. We have corrected this.

On page 15 line 318, the last one of the five modes should be unimodal with NOP.

Response: Thanks.

In Fig. S2, the titles of subfigures should be U(1 NOP).

Response: Thanks. We have corrected this.

On page 16 line 328-331, the authors discussed different scenarios that the evolution of the mRNA distribution may undergo. I would suggest to add more detailed discussion on the critical conditions or sensitive parameters giving rise to different scenarios and what biological insights can be obtained. What are the different conditions giving rise to results presented in S2 and S3?

Response: From Fig. 4 and SI Figs. S2-3, we see that the peaks are more sensitive to the change of d_G when k_EP is small, but to the change of k_EP when d_G is large. Mathematically, the parameter Θ in the MB distribution are actually regulated jointly by k_EP and k_NN/d_G. When d_G is larger, k_EP is relatively larger compared to k_NN/d_G if k_NN is fixed, and hence occupies

a dominant position. This implies that k_EP is more sensitive to Θ, and further implies that the peaks are more sensitive to k_EP. This sensitivity phenomenon indicates that **over long genomic distances, the long-range E-P interactions (parameter k_EP in our model) play an important role** in maintaining their normal communication and regulating gene expression. In addition, we investigate the effects of the E-P interaction strengths and friction coefficient on the pattern of mRNA distribution, finding the friction coefficient is non-sensitive (Additional Figure **4A**).

Considering that there are many downstream parameters, we change the minimum promoter activation rate (alpha_min) and minimum transcription initiation rates (mu_min), as well as the ratios of the maximum rate over the minimum rate (alpha_max/alpha_min, mu_max/mu_min), and then investigate impacts of the E-P interaction strengths and the ratios on the pattern of mRNA distribution (Additional Figure **4B-C**). We find that when the ratio mu_max/mu_min is large, that is, when the difference between the mu_max and mu_min is relatively large, the distribution of mRNA exhibits multimodality (Only a little small region has tirmodal, so we merge the bimodal and trimodal regions into multimodal regions) in a wide range. But at the same time, there is also an appropriate k_EP range that makes it possible to have two peaks even when the ratio is small. In fact, when **there is a relatively large gap between the mu_max and mu_min, the rate distribution may exhibit bimodal, leading to the bimodal of gene expression.** This may help us understand the transcription process and the formation of bimodal. The following Additional Figure **4C** shows the opposite result. In addition, the conditions for producing different results in Figs. S2 and S3 are mainly due to the different scales of upstream and downstream and the ratios of the maximum rate over the minimum rate.

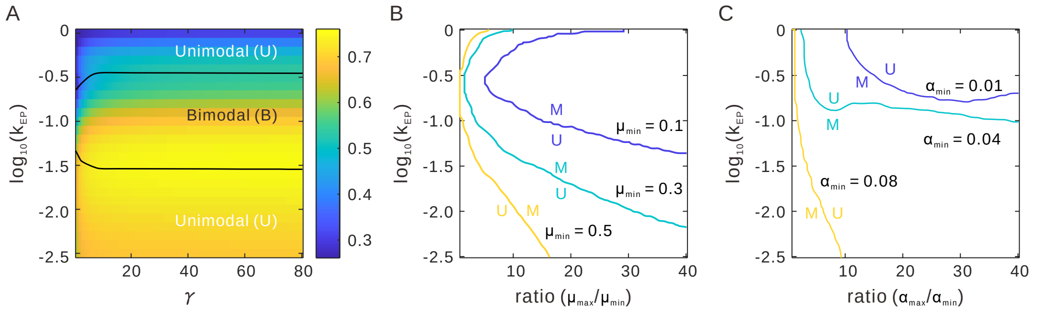

Additional Figure 4. The effects of parameter's changes on the multimodality of mRNA distribution. (A) Effects of E-P interaction strengths k_{EP} and friction coefficient γ on the pattern of mRNA distribution. Parameters are $d_G = 60$, $\alpha_{max} = 5$, $\alpha_{min} = 0.3$, $\beta = 0.5$, $\mu_{max} = 8$, $\mu_{min} = 2$, $\delta = 0.1$. (B) Effects of k_{EP} and the ratio of μ_{max}/μ_{min} on the pattern of mRNA distribution. The $\mu_{min} = 0.1,\ 0.3,\ 0.5$. Parameters are $\alpha_{max} = 0.10$, $\alpha_{min} = 0.01$. (C) Effects of k_{EP} and the ratio of $\alpha_{max}/\alpha_{min}$ on the pattern of mRNA distribution. The $\alpha_{min} = 0.01,\ 0.04,\ 0.08$. Parameters are $\mu_{max} = 5$, $\mu_{min} = 2$. Each line divides the entire region into two parts, with U indicating a single peak area and

M indicating a multimodal area. Other parameter values are set as $d_G = 60$, $\beta = 0.03$, $\delta = 0.1$, $\gamma = 1$. The solid line divides the entire region into two parts, with the part marked U indicating unimodal and the part marked M indicating multimodal.

Finally, we point out that since the system has many parameters, including upstream and downstream scale, **which would collectively regulate the resulting mRNA distribution**, it seems difficult to find the critical conditions giving rise to different scenarios, and large-scale sampling in high-dimensional parameters space is needed. However, we are only interested in effects of the parameters related to E-P communication on mRNA distribution, and find a consistent pattern of modulating mRNA distribution across parameter changes, as shown in our main text and Supplementary Text.

On page 17 line 355, different values of d_G were chosen by fitting the experimental data. This information should be provided to clarify how those values were obtained.

Response: Thanks for your suggestion. In Zuin's paper, the author provided E-P genomic distances for different cell lines. They are 112.1710 kb, 39.4530 kb, 23.1110 kb, 17.0190 kb and 6.0600 kb. We supposed that one monomer in our coarse-grained polymer model represented 5kb, and then we obtained corresponding d_Gs. We have added this information in the main text.

Grammar mistakes:

Page 10 line 205, mothod -> method

Page 11 line 223, challenge-> challenging

Page 11 line 236, or a a frozen E-P -> or a frozen E-P

Page 13 line 260, given vs -> given as

Page 14 line 285, CV downregulated by -> CV is downregulated by

Page 14 line 297, the mRNA level decrease and trends -> the mRNA level decreases and tends

Page 16 line 329, it should be ‘still changes from unimodal to the bimodal and then to unimodal again’.

Response: Thanks for your careful review. We have made corrections.
